# The complete mitochondrial genome of a cave-dwelling loach *Triplophysa baotianensis* (Teleostei: Nemacheilidae)

**DOI:** 10.1080/23802359.2021.1899861

**Published:** 2021-03-26

**Authors:** Yali Wang, Ning Xiao, Siwei Wang, Tao Luo, Xu Yang, Tao Liu, Jiang Zhou

**Affiliations:** a School of Life Science, Guizhou Normal University, Guiyang, Guizhou, PR China; b Guiyang Nursing Vocational College, Guiyang, Guizhou, PR China; c School of Karst Sciences, Guizhou Normal University, Guiyang, Guizhou, PR China

**Keywords:** Mitochondrial genome, phylogenetic tree, *Triplophysa baotianensis*

## Abstract

*Triplophysa baotianensis* belong to the genus *Triplophysa* (Teleostei, Nemacheilidae), endemic to Guizhou Province, Southwestern China. In this study, the complete mitochondrial genome of *T. baotianensis* was sequenced and reported for the first time. The circular mitogenome was 16,576 bp in length and consisted of 13 protein-coding genes, 2 ribosomal RNA (rRNA) genes, 22 transfer RNA (tRNA) genes, and 1 non-coding control region. The overall base composition was 30.79% A, 27.62% T, 25.46% C, and 16.13% G with 41.59% GC content. Phylogenetic analysis using mitochondrial genomes of 40 species showed that all *Triplophysa* species clustered as one monophyletic clade, and *T. baotianensis* was the closest to (*T. nasobarbatula* + (*T. rosa + T. xiangxiensis*)).

The genus *Triplophysa* Rendahl is a species-rich group in the family Nemacheilidae (Zhu 1989; Zhang et al. 2015). To date, nearly 180 species of *Triplophysa* have been described worldwide, of which, more than 100 species are distributed in China (Zhang et al. 2015; Eschmeyer et al. 2020). In China, species in the genus are primarily known to occur in the upper and middle portions of the Yangtze, Yellow River, Red River, and Pearl River drainages (Zhu 1989; He et al. 2012; Ren et al. 2012; Zhang et al. 2015). The genus includes some cave taxa species, which are distributed in karst areas with well-developed limestone caves and underground rivers, such as those in Guangxi, Guizhou, Yunnan, and adjacent regions characterized by a typical karst environment in South China. The *Triplophysa baotianensis* (Li, Liu, Li & Li, 2018) is a species described on the basis of specimens collected from Baotian Town, Panzhou city, Guizhou Province, China and is a typical cave-dwelling fish. The mitochondrial genome sequencing of this species is of great importance. First, the *T. baotianensis* is a recently published species with only morphological characters and lacking molecular data. Second, the species originated from the upper Yangtze River and may be the ancestor of the cave taxon, which needs further study.

The specimens of *T. baotianensis* were collected from the Nanpanjiang River (25°24′28.54″N, 104°43′11.87″E), Panzhou City, Guzihou Province, China. After morphological identification, the specimens were stored in 95% alcohol at −20 °C. Total DNA was extracted from the muscle of the dissected walking legs of the specimen using the CTAB method. Then, for each sample, the DNA fragments were mechanically interrupted (ultrasound) into small fragments (∼350 bp), and the DNA fragments with prominent ends were repaired using a combination of 3′5′nucleic acid exonuclease and polymerase, with ‘A’ added to the last 3′-end, and the sequencing joints were connected and PCR amplified to form sequencing libraries. The merged DNA was used to construct the sequencing library using the NEBNext^®^Ultra™DNA Library Prep Kit and sequenced on an Illumina NovaSeq 6000 sequencer. Each sample generated approximately 4.1 GB of 150-bp raw data. These reads sequences were bioinformatically sorted by barcode sequence and assembled into mitochondrial genomes using SPAdes version 3.14.0 (Bankevichet al. 2012). After DNA sequencing, the complete mitochondrial genome of *T. baotianensis* was assembled and annotated and has been deposited in GenBank database under the accession number MT992550. The specimen was stored in the Animal Ecology Laboratory of the School of Karst Sciences (GZNU20180421005), Guizhou Normal University, Guiyang City, Guzihou Province, China.

The mitogenome of *T. baotianensis* was 16,576 bp in length and encodes 37 genes, included 13 protein-coding genes (PCGs), 2 ribosomal RNA (12S rRNA and 16S rRNA), 22 transfer RNA genes (tRNAs), 1 mitochondrial control region (CR or D-loop), and 1 non-coding region (NC). The base composition was 30.79% A, 27.62% T, 25.46% C, and 16.13% G, demonstrated a bias of higher AT content (58.41%) than GC content (41.59%). Among these genes, the ND6 gene and eight tRNA genes (*tRNA^Gln^*, *tRNA^Ala^*, *tRNA^Asn^*, *tRNA^Cys^*, *tRNA^Tyr^*, *tRNA^Ser^*^(UCN)^, *tRNA^Glu^*, and *tRNA^Pro^*) were encoded on theL-strand, the remaining genes were encoded on the H-strand. Most PCGs were initiated with a common ATG start codon, while COI utilized GTG as start codon, which was similar to other *Triplophysa* fishes (Wang et al. 2016). Four types of stop codons were observed in the PCGs, including TAA for ND1, COI, ATP8, ATP6, ND4L, ND5, and ND6, TAG for ND2 and ND3, T(AA)for COII and CYTB, TA(A) for COIII and ND4. Eleven genes overlap and 14 intergenic spacers were observed, with the total length of 34 and 61 bp, respectively.

Based on the concatenated 13PCG sequences, the phylogenetic relationship among *Triplophysa* species was reconstructed by using the Bayesian inference (BI) and maximum likelihood (ML) methods through MrBayes version 3.2.1 (Ronquist et al. 2012) and IQTREE version 2.0.4 (Nguyen et al. 2015). Both the ML and BI phylogenetic trees showed an identical topology (Figure 1). All the *Triplophysa* species were clustered together and then grouped with two *Barbatula* species with high bootstrap values (UBP/BPP = 100%/1.00), and similar results have been reported in other studies (Wang et al. 2016). Within the genus *Triplophysa*, *T. baotianensis* is closely related to (*T. nasobarbatula* + (*T. rosa + T. xiangxiensis*)) which is mainly distributed in the upper reaches of the Pearl River and Yangtze River, respectively. This newly sequenced complete mitogenome would contribute to further investigations of molecular evolution of genus *Triplophysa*, particularly the taxa of the genus that cave dwelling.

**Figure 1.**
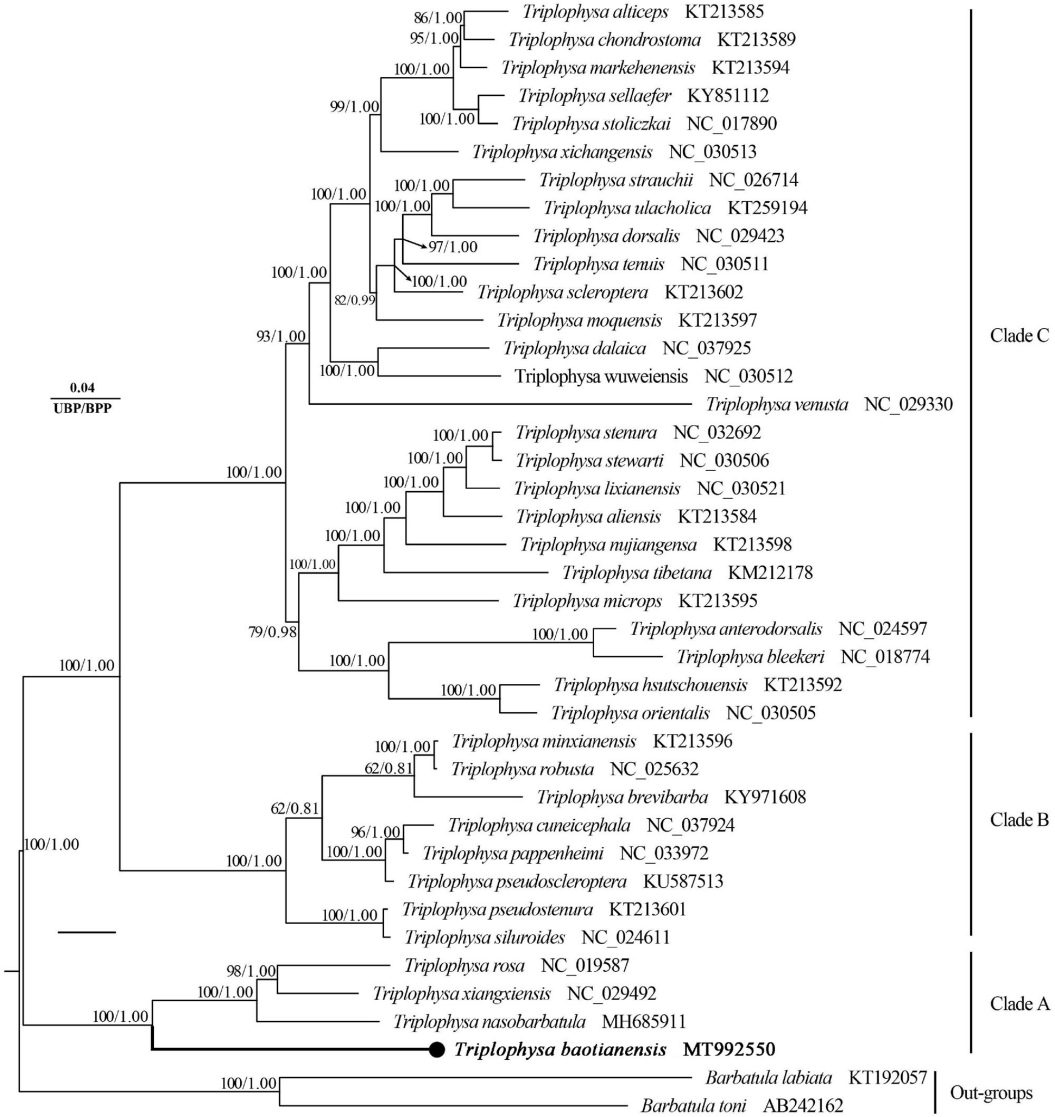
The phylogenetic tree inferred by the BI and ML method, based on 13 protein-coding genes from 40 species. Ultrafast bootstrap supports (UFB) from ML analyses/Bayesian posterior probabilities (BPP) from BI analyses were noted beside nodes.

## Data Availability

The data that support the findings of this study are openly available in NCBI at https://www.ncbi.nlm.nih.gov/nuccore/MT992550, Associated BioProject, https://www.ncbi.nlm.nih.gov/bioproject/PRJNA681252, BioSample accession number at SAMN16947108 and Sequence Read Archive at https://www.ncbi.nlm.nih.gov/sra/SRR13161315 or available from the corresponding author.
